# Comparison of Mineral Levels in Blood and Hair Samples of Healthy Adults: Evaluating the Clinical Utility of Hair Mineral Analysis

**DOI:** 10.1007/s12011-025-04793-w

**Published:** 2025-11-23

**Authors:** Alex Shahverdian, Simin Torabzadeh, David Watts, Steffen Porwollik, Monica Padilla, Jeong Su Lee, Gabriella Perez, Mahtab Jafari

**Affiliations:** 1 Department of Pharmaceutical Sciences, University of California, Irvine, Irvine, CA USA; 2 Department of Medicine, General Internal Medicine, University of California, Irvine, Irvine, CA USA; 3 Trace Elements, Incorporated, Addison, TX USA

**Keywords:** Hair mineral analysis, Blood mineral analysis, Mineral imbalance, Healthcare, Medication

## Abstract

Minerals play vital roles in human physiology and immunity. Their tightly regulated levels in the human body can be easily measured in blood, but that only provides a snapshot of the mineral status at the time of collection. In contrast, human hair accumulates minerals over time and is less invasive to collect and store. This pilot study compared mineral levels in blood and scalp hair of generally healthy adults to determine whether hair can accurately detect clinically relevant mineral imbalances and evaluate whether a relationship exists between blood and hair measurements. Minerals tested included calcium, magnesium, sodium, potassium, copper, zinc, phosphorus, iron, and selenium. Correlation, linear regression, and agreement analyses were performed before and after statistical outlier removal. A total of 30 subjects (mean age: 69.8 years) participated in the study. Most minerals displayed no significant correlation between hair and blood values. However, a weak but significant inverse correlation between blood and hair was identified for phosphorus (*p* = 0.044 in correlation and linear regression analyses). Linear regression analysis before outlier removal also suggested a significant direct correlation of blood and hair values for zinc (*p* = 0.0315), but not after outlier removal (*p* = 0.2682). Agreement between mineral status in blood and hair (low, normal, high, based on established reference ranges) was non-significant for all minerals tested. In conclusion, a significant relationship between blood and hair mineral concentrations was not established. However, the distinct hair mineral analysis results suggest a unique clinical utility for long-term trends and imbalances.

**Supplementary Information:**

The online version contains supplementary material available at 10.1007/s12011-025-04793-w.

## Background & Introduction

Approximately 20 minerals are essential for human function, including metals such as sodium (Na), potassium (K), magnesium (Mg), calcium (Ca), iron (Fe), copper (Cu), and zinc (Zn) [1]. These minerals play a vital role in human physiology, including muscle and nerve function, fluid homeostasis, and bone development [2]. Some minerals are also crucial for both innate and adaptive immunity [3]. Zinc, for example, acts as an antioxidant to protect against reactive oxygen species while also playing a central role in cellular growth and differentiation of immune cells [2, 3]. Iron is involved in cytokine regulation, and copper affects T cell proliferation [2, 3]. Minerals also act as vital cofactors for enzymatic reactions [2, 3]. Selenium (Se)-dependent enzymes are important for leukocyte and natural killer cell function [3] and magnesium is a necessary cofactor for the synthesis and replication of DNA and RNA [2]. Additionally, iron-sulfur (Fe-S) protein clusters are essential cofactors in mediating electron transfer within the mitochondrial respiratory chain [4]. Regular monitoring of these minerals is important in clinical practice for the diagnosis, prevention, and treatment of various diseases. 

Blood and its cell-free form, serum, are the most commonly used biospecimens in clinical practice for the early detection and treatment of mineral imbalance [5, 6] and provide a snapshot of a patient’s mineral status at the time of collection [7]. Routine testing and monitoring of minerals in blood samples has an important role in identifying acute imbalances that can result in serious complications such as arrhythmias, seizures, and even death [8, 9]. However, blood mineral levels do not indicate whether mineral imbalances have been ongoing over an extended period of time. Moreover, mineral concentrations in blood are the result of homeostatic mechanisms and may not correspond to the mineral content of the whole individual [10].

Hair samples may accumulate minerals over weeks to years, and may contain higher mineral concentrations compared to blood [7, 11, 12]. As hair draws in minerals from the bloodstream, they are permanently incorporated into its keratin structure [10]. Hair has other advantages over blood, such as its non-invasive collection methodology, higher stability during storage and transport, and fewer hazards [7, 11, 12]. Therefore, hair analysis has been widely used for detecting heavy metals, especially in populations prone to occupational or environmental exposure. Research into its application for detecting and monitoring mineral deficiencies and excesses is also expanding [11–13].

Several studies have compared hair mineral concentrations in subjects suffering from various health conditions, such as hypertension, diabetes, anemia, obesity, liver cirrhosis, cancer, Alzheimer’s disease, and inflammatory bowel disease, to those of healthy individuals (Table 1). These studies investigated the role that mineral imbalance may have played in these conditions and included hair samples, in addition to blood, to better understand the overall mineral status in the studied populations. One such study failed to find a significant difference in the hair and serum levels of zinc, iron, and copper between females with and without alopecia areata but determined a significant correlation between serum and hair levels of these three minerals in their study subjects [14].

**Table 1:** Studies evaluating the utility of hair mineral analysis in human diseases

Minerals Analyzed in Study	Study Population (Numbers of individuals)	Main Results	Reference
Ca, K, Mg, Na	Healthy (182), hypertensive diabetic (254), non-hypertensive diabetic (228)	Mean K, Mg, and Ca levels were lower in all subjects with diabetes (hypertensive and non-hypertensive) in both blood and hair samples, compared to controls. Na levels were higher in the blood and hair of hypertensive and non-hypertensive diabetic patients, compared to controls.	[41]
Cd, Cu, Cr, Fe, Ni, Pb, Zn	Non-anemic (134), anemic (132)	Lower levels of Fe, Cu, and Zn in biological samples of subjects with anemia compared to controls. Pb and Cd were significantly higher in children with anemia in all age groups.	[42]
Ca, Cu, Fe, Mg, Zn	Healthy (40), obese/hypertensive/insulin-resistant (40)	Higher serum Fe, higher serum and hair Ca, and lower hair Zn levels were found in the study group. A significant correlation between serum and hair concentrations of selected minerals was observed.	[43]
As, Cd, Se, Zn	Healthy female subjects (75), liver cirrhotic female subjects (40), female patients with liver cancer (92)	Levels of Se and Zn were lower in subjects with liver cirrhosis or liver cancer compared to those of healthy females. Levels of As and Cd were two to three-fold higher in subjects with liver cirrhosis or liver cancer, compared to healthy individuals. After mineral supplementation, lower incidences of Zn and Se deficiency were shown.	[44]
Cu, Zn	Healthy (100), androgenetic alopecia (116)	Subjects showed decreased Cu and Zn levels in hair, whereas no changes in serum and urine were found.	[45]
Zn, Cu, Fe	Healthy female subjects (27), female patients with alopecia areata (16)	There was no statistical difference in mineral levels of Zn, Fe, and Cu between subjects with alopecia areata and healthy individuals.	[14]
Cu, Fe, Mg, Mn, Se, Zn	Healthy (33), Alzheimer's disease (45)	Lower hair Se and Zn levels, higher Cu and Mn levels in subjects with Alzheimer's disease, compared to healthy subjects (*p* < 0.05). No significant difference between the two groups in Mg and Fe levels (*p* > 0.05). No significant difference in hair mineral analysis vs serum in Cu, Se, Zn, Mg, Mn, and Fe.	[46]
Ca, P, Mg, Fe, Zn, Se	Healthy (29), inflammatory bowel disease (65)	Significant difference between hair Fe, Se, and Mn levels of the two groups (*p* < 0.05). Significant difference between serum Ca, Fe, Zn, and Se (*p* < 0.05).	[47]
Fe, Zn, Cu	Healthy (31), type 2 diabetes (31)	Subjects with diabetes had high dietary Fe intake, higher hair Fe, and lower serum Zn levels than subjects without diabetes. Hair Zn and Cu levels were comparable in both groups.	[48]
Al, As, Ca, Cd, Co, Cr, Cu, Fe, Hg, I, Mg, Mn, Ni, Se, V, Zn	Autism spectrum disorder (ASD) with catatonia (30), ASD without catatonia (30), healthy (30)	Hair Ca and Se levels were lower, and hair Hg levels were higher in subjects with ASD vs controls. Subjects with ASD with catatonia had elevated serum Cr, Cu, and V compared to controls.	[49]
Ca, Cu, Mg, Se, Zn	Healthy (66), chronic rhinosinusitis (88)	Blood Ca, Mg, Se, Zn, and hair Ca, Cu, Mg, and Zn levels in subjects with chronic rhinosinusitis were lower than in controls.	[50]
Ca, Cu, Fe, Mg, Se, V, Zn	Normal weight (40), obese (40)	Serum Ca, Fe, Mg, Se, V, Zn levels, hair Fe, Mg, V content, and urinary Se and V concentrations were lower in subjects with obesity compared to controls.	[51]

Although hair analysis is a less invasive test in clinical practice, it has some limitations, including a higher chance of contamination, a lack of standardized procedures, and high variability between laboratories [7, 15]. Moreover, reference ranges for mineral concentrations in hair samples are not universally established, and hair mineral testing laboratories often set their own reference ranges [16]. The Clinical Laboratory Improvement Amendments (CLIA) of 1988 set regulations for all facilities in the United States that test human specimens for health or disease assessment [17, 18]. Although some hair analysis assays are CLIA certified, these assays are limited to testing drugs and environmental toxins, not to testing minerals.

Limited research has been conducted on the comparative analysis of hair and blood mineral concentrations within a single cohort to investigate potential relationships [14, 19–21]. These existing studies are either several decades old [19, 20] or have focused on specific patient populations and diseases [14, 19–21]. With the growing popularity of hair as a biospecimen, more studies are needed to explore its relationship with blood mineral levels. We therefore compared mineral levels in blood and hair from the same individuals to determine whether hair can accurately detect clinically relevant mineral imbalances and to evaluate if a relationship exists between hair and blood mineral measurements.

## Methods

We conducted a pilot, prospective, single-center, within-subject clinical study to compare and evaluate the levels of selected minerals in blood (serum) and hair samples of the same individuals. The minerals included calcium, magnesium, sodium, potassium, copper, zinc, phosphorus, iron, and selenium.

### Subject Recruitment

Subjects were recruited from a University of California, Irvine (UC Irvine) Health Primary Care clinic in Irvine, California. Participating individuals were 50 years of age or older who had a scalp hair length of at least 1.5 inches, were generally healthy, and were under the care of a primary care physician at UC Irvine Health. The scalp hair was required to have remained undyed, unpermed, and unbleached for at least a month before sample collection time. Individuals who were pregnant or breastfeeding or were occupationally exposed to external contaminants, such as during welding or mining, were excluded from the study. The age requirement was chosen due to the increased incidence of mineral abnormalities that come with age [22]. For the purpose of this study, “generally healthy” volunteers included ambulatory subjects who considered themselves in good health, whose chronic illnesses were well managed, and who did not have acute disease.

A total of 30 subjects completed the study. Subjects answered a questionnaire that included demographic information, lifestyle habits, medical conditions, prescribed medications, and current supplements. A summary of their demographic data is shown in Table 2.

**Table 2:** Study subjects’ demographics

Characteristics	
Age (years)	
Mean	69.8
Female, mean	69.3
Male, mean	71.1
Range	52–86
Gender (*n*)	
Female	23 (77%)
Male	7 (23%)
Other	0
Ethnicity/Race (*n*)	
Caucasian	25 (83%)
Asian	2 (7%)
Hispanic/Latino	2 (7%)
Middle Eastern	1 (3%)
Weight (lbs)	
Mean	156.25
Female, mean	154
Male, mean	163.6
Height	
Mean	5 ft. 5 in
Female, mean	5 ft. 4 in
Male, mean	5 ft. 9 in
BMI (kg/m^2^)	
Mean	25.7
Female, mean	26.4
Male, mean	24.2

## Blood and Hair Collection and Analysis

Consenting subjects attended a single visit to the UC Irvine Center for Clinical Research (Irvine, CA, USA), where both hair and blood samples were obtained. Hair samples were placed in a clean hair specimen envelope and sent to Trace Elements, Inc. (Addison, TX, USA) for analysis. Blood samples were centrifuged and sent to UCI Medical Center Pathology & Laboratory Services (Orange, CA, USA) for analysis.

## Hair Collection and Analysis

Subjects were instructed to wash their hair as they normally would prior to attending their clinic appointment. Subjects were also asked not to apply any hair products after showering and before their clinic visit, and were required to have discernible, undyed, unpermed, and unbleached scalp hair visible from the root at the time of collection. Using high-grade stainless-steel scissors, hair samples were cut from four to five different scalp locations that included the nape of the neck, the posterior vertex, and posterior temporal regions. Cuts were made as close to the root as possible, and only the proximal inch of hair was retained. We acknowledge that this segment of hair does not temporally correspond to the mineral concentrations in blood at the time of drawing; however, it represents the standard sampling location for hair mineral analysis [23, 24]. Any excess hair beyond this length was discarded. Approximately 100 mg of hair was collected from each subject using a standard hair weight scale card and placed in a clean hair specimen envelope to be sent for analysis.

Results were reported as milligrams of mineral percent (mg/100g). State-of-the-art inductively coupled plasma mass spectrometry (ICPMS) and uniform temperature-controlled microwave digestion techniques were utilized for all current reference range studies from an initial database consisting of 2,200 subjects. Determination of reference ranges by Trace Elements, Inc. was based on a selection of apparently "healthy" individuals. Reference ranges were as follows: calcium (22–104 mg/100g), magnesium (1.3–9.4 mg/100g), sodium (3–34 mg/100g), potassium (2–23 mg/100g), copper (0.9–3.2 mg/100g), zinc (9–22 mg/100g), phosphorus (10–21 mg/100g), iron (0.5–2.0 mg/100g), and selenium (0.04–0.12 mg/100g).

Hair mineral measurements were performed on a Perkin-Elmer mass spectrometer (Perkin-Elmer NexION). The accuracy of each calibration and the entire methodology was verified by reagent blanks, independent calibration verification standard solutions, pooled hair specimens, pooled hair aqueous solutions, and other certified hair reference materials with established reference ranges.

## Blood Collection and Analysis

Approximately 32 mL of blood were collected from each subject. Subjects were not instructed to follow any dietary restrictions or to discontinue medications or supplements during the study. Standard laboratory procedures were used for analysis, including o-cresolphthalein complexone-end for calcium, 2,4,6-tripyridyl-s-triazine-end for iron, ion-selective electrode (ISE)-crown-ether membrane for potassium and sodium, xylidyl blue for magnesium, phosphomolybdate-end for phosphorus, and ICPMS for copper, zinc, and selenium. Reference ranges were set by UCI Health Pathology & Laboratory Services, as follows: calcium (8.6–10.3 mg/dL), copper (male: 70–140 mcg/dL, female: 85–155 mcg/dL), iron (male: 49–181 mcg/dL, female: 37–170 mcg/dL), potassium (3.5–5.1 mmol/L), magnesium (1.9–2.7 mg/dL), sodium (136–145 mmol/L), phosphorus (2.5–5.0 mg/dL), selenium (23–190 mcg/L), and zinc (55–150 mcg/dL).

### Statistical Analysis

Statistical analysis was performed with GraphPad Prism (GraphPad Software, Boston, MA) version 10.3.0. Blood and hair values for each mineral were tested for normality using a D'Agostino-Pearson test (alpha = 0.05). If both specimens of a subject passed normality for the mineral, correlation was evaluated with a Pearson’s correlation test (alpha = 0.05). A Spearman’s correlation test (alpha = 0.05) was used if the blood or the hair mineral measurements did not pass normality. Simple linear regression was also performed between the two biospecimens (confidence interval = 95%).

Outlier detection was performed using the *Robust Regression and Outlier Removal* (ROUT) method with a false discovery rate (Q) set at 1% to minimize false-positive outlier identification [25]. The ROUT method is used for datasets with potential non-Gaussian distributions and was applied to each analyte individually using GraphPad Prism (version 10.3.0). If a hair mineral measurement was identified as an outlier by the ROUT analysis, both hair and blood results from the subject were removed from the analysis.

A Cohen’s kappa analysis, a chance-corrected method for assessing agreement, evaluated what proportion of values were in agreement between blood and hair [26]. Analysis was conducted on GraphPad after classifying the obtained mineral content results as low, normal, or high, based on the reference ranges in each specimen (confidence interval = 95%).

## Results 

### Global Blood and Hair Mineral Analysis Results

The mineral concentrations in blood and hair samples from all 30 individuals are displayed in Supplementary Table 1. Overall, 264 paired blood/hair mineral measurements (PBHMMs) were taken (30 each for Ca, Cu, Fe, K, Mg, Na, Se, and Zn; 24 for phosphorus, P). Measurements within established specific standard ranges for each mineral were classified as “normal” (N), whereas those outside specific reference ranges were either “high” (H) or “low” (L). A total of 21 subjects had at least one hair mineral value outside the standard range, and 12 subjects had at least one such blood mineral value.

For 212 of 264 PBHMMs (80.3%), blood and hair mineral concentrations were both within the standard ranges established for each mineral. The remaining 52 PBHMMs had a variety of relationships. Normal blood values were accompanied by high hair mineral concentrations in 22 PBHMMs (4 Ca, 4 K, 5 Mg, 5 Na, 2 Se, and 2 Zn) and by low hair mineral concentrations in 13 PBHMMs (1 Ca, 6 Cu, 5 K, and 1 Mg). Conversely, normal hair measurements were paired with high blood values in 5 PBHMMs (2 Ca, and 3 K), and with low blood values in 8 PBHMMs (1 Cu, 1 K, 4 Mg, and 2 Na). In 3 PBHMMs, subjects with a low blood mineral concentration had a high hair concentration (1 Ca, and 2 K). Finally, one Cu PBHMM displayed low values for both measurements. The most measurements outside the standard range were recorded for K (6 of 30 blood samples, 11 of 30 hair samples, Table 3), while all Zn and Fe values were within the established standard ranges.

**Table 3:** Overview of mineral concentrations in blood and hair

	Ca		Cu		Fe		K		Mg		Na		P*		Se		Zn	
	Blood	Hair	Blood	Hair	Blood	Hair	Blood	Hair	Blood	Hair	Blood	Hair	Blood	Hair	Blood	Hair	Blood	Hair
Normal Range	8.6–10.3 mg/dL	22–104 mg/100 g	Male: 70–140 mcg/dL, Female: 85–155 mcg/dL	0.9–3.2 mg/100 g	Male: 49–181 mcg/dL, Female: 37–170 mcg/dL	0.5–2.0 mg/100 g	3.5–5.1 mmol/L	2–23 mg/100 g	1.9–2.7 mg/dL	1.3–9.4 mg/100 g	136–145 mmol/L	3–34 mg/100 g	2.5–5.0 mg/dL	10–21 mg/100 g	23–190 mcg/L	0.04–0.12 mg/100 g	55–150 mcg/dL	9–22 mg/100 g
Number of measurements within the normal range	27	24	28	23	30	30	24	19	26	24	28	25	24	24	30	28	30	28
Number of measurements outside the normal range [Low / High]	0 / 3	2 / 4	2 / 0	7 / 0	0 / 0	0 / 0	1 / 5	7 / 4	4 / 0	1 / 5	2 / 0	0 / 5	0 / 0	0 / 0	0 / 0	0 / 2	0 / 0	0 / 2
Number of ROUT outliers [Low / High]	N/A	0 / 1	N/A	0 / 2	N/A	0 / 1	N/A	0 / 5	N/A	0 / 1	N/A	0 / 6	N/A	0 / 0	N /A	0 / 2	N/A	0 / 1
ROUT outliers	N/A	296 mg/100 g	N/A	2.3, 2.4 mg/100 g	N/A	1.5 mg/100 g	N/A	32, 75, 47, 166, 15 mg/100 g	N/A	23.9 mg/100 g	N/A	32, 39, 37, 92, 108, 72 mg/100 g	N/A	N/A	N/A	0.98, 3.24 mg/100 g	N/A	33 mg/100 g

* *n* = 24

Statistical outliers in hair mineral concentrations, as identified by ROUT analysis, were detected for all minerals except phosphorus. Na exhibited the highest number of ROUT-identified outliers, with 6 of 30 values deviating from the normal distribution (Table 3, see Supplementary Table 1 for details of all ROUT-identified outliers). Note that the mineral concentrations of ROUT-identified outliers were often but not always outside the standard ranges. Overall, ROUT analysis identified 13 samples as outliers. We were unable to correlate specific hair care routines and products with specific mineral measurement aberrations.

### Notable Hair Mineral Concentration Aberrations

Several subjects demonstrated markedly elevated or multiple abnormal hair mineral concentrations accompanied by largely normal blood mineral measurements, prompting further investigation into prescription medications, over-the-counter drugs, dietary supplements, or hair care products that may explain these aberrations. As an example, subject 2, a 52-year-old female, had elevated selenium levels in her hair (3.24 mg/100g, reference range [RR]: 0.04–0.12 mg/100g), while also displaying elevated calcium (123 mg/100g, RR: 22–104 mg/100g) and magnesium (13.4 mg/100g, RR: 1.3–9.4 mg/100g) hair levels, with reduced potassium levels (1 mg/100g, RR: 2–23 mg/100g). All blood mineral concentrations were within normal limits. The subject did not take any prescription medications. However, she used the "blue version" of Head & Shoulders shampoo, potentially a clinical-strength product variety that contains 1% selenium sulfide, an active ingredient used in dandruff treatment [27]. She also reported daily use of an anti-aging dietary supplement (SeroVital®), which contains an undisclosed amount of magnesium stearate, a common lubricant in tablet formulations [28]. These observations may explain, in part, the elevated Se and Mg levels measured in this subject’s hair.

One other subject displayed elevated selenium levels in his hair, subject 1, an 81-year-old male (0.98 mg/100g, RR: 0.04–0.12 mg/100g). The only blood mineral outside the normal limit in this subject was potassium (5.2 mmol/L, RR: 3.5–5.1 mmol/L). Subject 1 took multiple prescription medications (apixaban, carvedilol, rosuvastatin, omeprazole) and vitamin supplements, and did not report using a specific shampoo. The reason for the elevated selenium level in his hair remains unknown.

Subject 17, a 64-year-old female, had elevated calcium (296 mg/100g, RR: 22–104 mg/100g) and magnesium (23.9 mg/100g, RR: 1.3–9.4 mg/100g) levels with reduced potassium (1 mg/100g, RR: 2–23 mg/100g) levels in her hair. Blood mineral levels were within normal limits. The subject did not use any prescription medications but reported regular consumption of 10,000 IU of vitamin D weekly, well above the 600 IU recommended dietary allowance (RDA) per day [29], and 250 mg of magnesium oxide (RDA: 320 mg/day [30]) five times per week. Vitamin D has been shown to cause hypercalcemia, which may explain, at least in part, these findings [31]. The subject was also using Daeng Gi Meo Ri Ki shampoo, however, none of its ingredients could be linked to these elevations.

Subject 33, a 62-year-old female, had elevated potassium (75 mg/100g, RR: 2–23 mg/100g) and sodium (92 mg/100g, RR: 3–34 mg/100g) levels in her hair, with normal blood mineral levels. She was on daily prescription medications (rosuvastatin, levothyroxine) and took multivitamin preparations, a vitamin B complex supplement, omega-3 fish oil, melatonin, and over-the-counter ibuprofen. However, this medical history did not explain the observed elevated hair mineral levels. The subject was also using Redken Color Extend shampoo. Sodium and potassium compounds are often present in shampoos, including Redken Color Extend. A similar hair mineral profile was observed in two more study participants, subjects 39 and 43. Subject 39, a 79-year-old female (47 mg/100g K, RR: 2–23 mg/100g; 108 mg/100g Na, RR: 3–34 mg/100g), took benazepril and amlodipine daily for blood pressure management. Magnesium was the only blood mineral level outside of normal limits (1.6 mg/dL, RR: 1.9–2.7 mg/dL). ACE inhibitors, such as benazepril, have been shown to increase potassium levels in blood, which may, in turn, influence hair mineral levels [32]. However, as indicated above, elevated sodium and potassium levels in hair, in the absence of other aberrations, may also stem from hair care routines and products. This subject did not disclose her shampoo. Subject 43, a 77-year-old female (166 mg/100g K, RR: 2–23 mg/100g; 72 mg/100g Na, RR: 3–34 mg/100g) with normal blood mineral levels took rosuvastatin daily and denosumab, an osteoporosis medication, every six months. Notably, this subject used Nutrafol, a dietary supplement against hair loss. Nutrafol’s ingredients include 5 mg of sodium per serving in addition to ashwagandha and saw palmetto extracts. Moreover, this subject was using Johnson & Johnson Baby Shampoo, a product with multiple sodium compounds.

These examples illustrate how hair mineral analyses may prompt further clinical investigations in search of possible explanations for abnormal measurements. Note that this study did not collect nutritional data from subjects, which may have provided further possible reasons for aberrant hair mineral values. Although we have been unable to identify a relevant clinical disorder in our small dataset, such personalized efforts may identify syndromes that otherwise go undetected, as demonstrated in the past [33].

### Correlation Between Hair and Blood Mineral Levels

A summary of the blood-hair correlation analysis by Pearson or Spearman tests is shown in Table 4. Before ROUT-outlier removal, except for phosphorus, none of the minerals showed a statistically significant correlation between the two specimen types. Phosphorus, the exception, demonstrated a significant inverse correlation between blood and hair samples (*r* = −0.414; CI: −0.701, −0.013; *p *= 0.044). Removal of ROUT outliers did not result in statistically significant correlations for any of the other analyzed minerals.

**Table 4:** Correlation analysis between mineral content measurements in human blood and hair samples

Mineral	Number of samples	Analysis type	Correlation coefficient *r* before outlier removal (95% CI)	*p*-value (two-tailed) before outlier removal	Number of outliers*	Correlation coefficient *r* (95% CI) after outlier removal	*p*-value (two-tailed) after outlier removal
Calcium	30	Spearman	−0.135 (−0.481, 0.247)	0.477	1	−0.1832 (−0.5234, 0.2074)	0.341
Copper	30	Spearman	−0.049 (−0.411, 0.327)	0.798	2	0.01797 (−0.3676, 0.3982)	0.928
Iron	30	Spearman (before outlier removal) / Pearson (after outlier removal)	−0.118 (−0.468 0.263)	0.535	1	−0.1144 (−0.4616, 0.2631)	0.554
Potassium	30	Spearman	−0.259 (−0.574, 0.123)	0.168	5	−0.1751 (−0.5421, 0.2480)	0.402
Magnesium	30	Spearman	−0.060 (−0.421, 0.317)	0.752	1	−0.1605 (−0.5062, 0.2297)	0.406
Sodium	30	Spearman	0.077 (−0.301, 0.435)	0.686	6	−0.06324 (−0.4650,0.3601)	0.769
Selenium	30	Spearman	0.208 (−0.176, 0.536)	0.271	2	0.1584 (−0.2391, 0.5105)	0.421
Zinc	30	Spearman (before outlier removal) / Pearson (after outlier removal)	0.118 (−0.263, 0.468)	0.535	1	0.2126 (−0.1669, 0.5372)	0.268
Phosphorus	24	Pearson	−0.414 (−0.701, −0.013)	**0.044**	0	n/a	n/a

* Identified by the *robust regression and outlier removal* (ROUT) method, Q coefficient of 1%

**Fig. 1:**
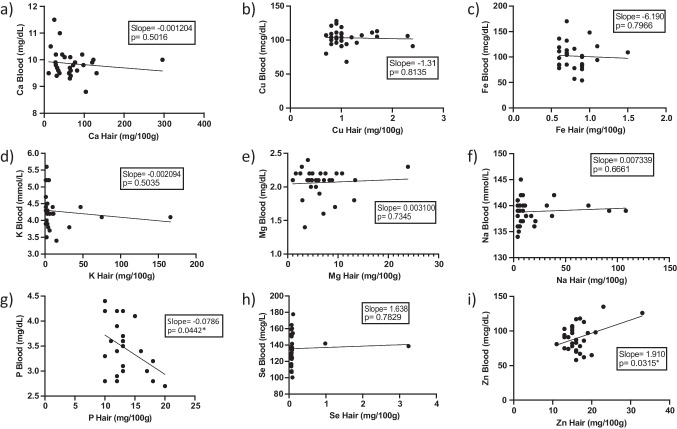
Linear regression analysis for nine minerals (**a**. calcium, **b**. copper, **c**. iron, **d**. potassium, **e**. magnesium, **f**. sodium, **g**. phosphorus, **h**. selenium, **i**. zinc) in human hair and blood specimens before outlier removal

**Fig. 2:**
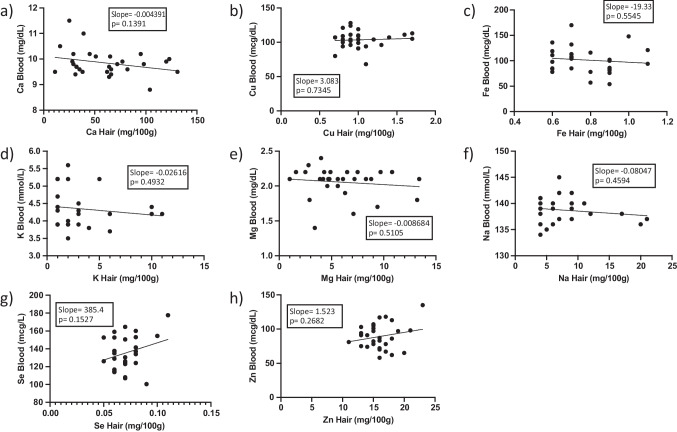
Linear regression analysis for eight minerals (**a**. calcium, **b**. copper, **c**. iron, **d**. potassium, **e**. magnesium, **f**. sodium, **g**. selenium, **h**. zinc) in human hair and blood specimens after outlier removal. No outliers were identified for phosphorus

Linear regression analysis results are summarized in Table 5**, **Figs. 1 and 2. Goodness of fit (*r*^2^) data were generally low for the minerals. However, linear regression analysis before ROUT outlier removal yielded statistically significant results for phosphorus and zinc. Specifically, phosphorus exhibited an inverse relationship between blood and hair concentrations (*r*^2^ = 0.1716, slope = −0.0786, *p *= 0.0442), confirming the correlation analysis results, while zinc showed a direct relationship (*r*^2^ = 0.1547, slope = 1.91, *p* = 0.0315). However, regression quality and significance for zinc decreased following ROUT outlier removal (Table 5).

**Table 5:** Linear regression analysis between mineral content measurements in human blood and hair samples

Mineral	Goodness of Fit (*r*^2^) before outlier removal	Slope before outlier removal	*p*-value before outlier removal	Number of outliers*	Goodness of Fit (*r*^2^) after outlier removal	Slope after outlier removal	*p*-value after outlier removal
Calcium	0.01628	−0.001204	0.5016	1	0.07924	−0.004391	0.1391
Copper	0.002022	−1.311	0.8135	2	0.0045	3.083	0.7345
Iron	0.002413	−6.19	0.7966	1	0.01309	−19.33	0.5545
Potassium	0.01614	−0.002094	0.5035	5	0.02064	−0.02616	0.4932
Magnesium	0.004173	0.0031	0.7345	1	0.01621	−0.008684	0.5105
Sodium	0.006748	0.007339	0.6661	6	0.02512	−0.08047	0.4594
Phosphorus	0.1716	−0.0786	**0.0442**	0	n/a	n/a	n/a
Selenium	0.002756	1.638	0.7829	2	0.07706	385.4	0.1527
Zinc	0.1547	1.91	**0.0315**	1	0.04519	1.523	0.2682

* Identified by the *robust regression and outlier removal* (ROUT) method, Q coefficient of 1%

The summary of the results of a kappa analysis of the agreement of the obtained data between human hair and blood measurements of minerals is shown in Table 6. Iron and phosphorus were excluded from the kappa analysis as all values were within reference ranges in the blood and hair specimens studied here. Observed agreement ranged from 50% for calcium to 93.33% for selenium and zinc before ROUT outlier removal, and from 60% for potassium to 96.55% for zinc after ROUT outlier removal. However, these observed agreements were attributed to chance as kappa was non-significant for all minerals included in the analysis.

**Table 6:** Kappa analysis for mineral measurements in human blood and hair samples

Mineral	Number of samples	Observed Agreement (%) before outlier removal	Kappa before outlier removal	95% CI of Kappa before outlier removal	Weighted Kappa before outlier removal	Number of outliers*	Observed Agreement (%) after outlier removal	Kappa after outlier removal	95% CI of Kappa after outlier removal	Weighted Kappa after outlier removal
Calcium	30	73.33	0	(−0.202, 0.202)	−0.098	1	75.86	0.024	(−0.197, 0.245)	−0.084
Copper	30	76.67	0.132	(−0.221, 0.485)	0.132	2	75	0.125	(−0.228, 0.478)	0.125
Potassium	30	50	−0.079	(−0.246, 0.088)	−0.118	5	60	0.057	(−0.120, 0.234)	0
Magnesium	30	66.67	−0.017	(−0.188, 0.154)	−0.031	1	68.97	−0.004	(−0.190, 0.182)	−0.036
Sodium	30	76.67	−0.050	(−0.100, 0.000)	0	6	91.67	0	(−0,0)	0
Selenium	30	93.33	0	(0, 0)	0	2	n/a**	n/a**	n/a**	n/a**
Zinc	30	93.33	0	(0, 0)	0	1	96.55	0	n/a***	0

* Identified by the *robust regression and outlier removal *(ROUT) method, Q coefficient of 1%

** After outlier removal, all selenium values were within normal limits

*** Undetermined

## Discussion & Conclusion

This study revealed no significant relationship between blood and hair mineral concentrations for the majority of the analyzed elements, indicating that mineral levels in one biospecimen cannot be reliably inferred from those in the other. Among the nine minerals, only phosphorus exhibited a statistically significant inverse correlation between blood and hair. In addition, our findings with zinc were mixed. Although zinc failed to demonstrate significance in correlation analyses and displayed a non-significant kappa, it reached significance in the linear regression analysis, indicating a direct relationship between blood and hair values (*r*^2^ = 0.1547, slope = 1.91, *p* = 0.0315). However, after ROUT outlier removal (subject 36, 33 mg/100g), this relationship was no longer significant (*r*^2^ = 0.04519, slope = 1.523, *p* = 0.2682).

Previous studies similarly report mixed results regarding zinc level relationships between blood and hair [14, 19, 20]. Two clinical studies involving contraceptive medications described no significant correlation of zinc, copper, magnesium, and iron concentrations between blood and hair in control and/or experimental groups [19, 20], which aligns with our findings. However, another study involving subjects with alopecia areata found a significant correlation between blood and hair for zinc, iron, and copper in study subjects and controls [14].

Phosphorus was the only mineral that demonstrated a statistically significant, albeit inverse, correlation and a linear relationship between blood and hair concentrations (correlation *r* = −0.414, CI: −0.701, −0.013, *p* = 0.044, linear regression *r*^2 ^= 0.1716, slope = −0.0786, *p* = 0.0442). We are unaware of previous studies that assessed the phosphorus level relationships between blood and hair within a single cohort. Based on our findings, further investigations are warranted to explore this relationship and its potential role in the incorporation of phosphorus into the keratinous structure of hair [10]. Note that in this study, the blood of three subjects was redrawn within one month after their initial clinic visit for phosphorus testing.

This pilot study examined the relationship between blood and hair mineral concentrations to assess the potential utility of hair mineral analysis in clinical practice. The lack of significant correlations in our data supports the concept that hair analysis provides a distinct, time-integrated assessment of an individual’s mineral status and not necessarily the mineral status on the collection day. This approach may help differentiate whether a single abnormal blood measurement reflects an underlying systemic condition or an acute physiological response. Notably, hair analysis has already demonstrated efficacy in detecting zinc deficiency in chronically malnourished children, highlighting its potential advantages over blood-based assessments in specific clinical contexts [33].

Our study highlighted the discrepancies observed between blood and hair mineral concentrations. Although not definitive, the use of medications, supplements, and shampoos may have contributed to the few abnormal mineral levels measured in hair samples. However, shampoo treatments usually have a negligible effect on test outcome, except products formulated with selenium sulfide, which has been shown to introduce selenium into hair structures [34].

The hair samples were not washed for the present study due to previous evidence of significant removal of endogenous elements with different washing techniques, where over 90% of the sodium and potassium were removed from hair by washing [35]. In this study, three subjects (subjects 33, 39, and 43) had elevated levels of sodium and potassium in their hair but normal blood concentrations of these minerals. While hair care routines cannot be ruled out as a contributing factor for these elevated mineral levels, two of these subjects were receiving rosuvastatin for the management of dyslipidemia, while the third subject was on antihypertensive medications, suggesting an elevated risk for cardiovascular disease. A study of 137 Korean individuals aged 60 to 79 years found significantly lower mean hair sodium concentrations in the low-risk atherosclerotic cardiovascular disease (ASCVD) group compared to those in the intermediate and high-risk groups (10 year ASCVD risk assessment; low, ≤ 5%; intermediate, 5% to < 7.5%; high, ≥ 7.5%, *p* = 0.004), although the differences between the groups did not reach statistical significance for potassium (*p* = 0.059) [36]. Patients with similar cardiovascular predispositions as these three subjects could benefit from using sodium hair analysis as a screening tool for ASCVD risk, where higher levels would warrant further diagnostic follow-up.

Given the pilot nature of this study, certain limitations must be acknowledged, including the small sample size (30 participants) and the overrepresentation of women (77%) and individuals of Caucasian ethnicity (83%). Future studies with larger, more diverse cohorts are necessary to ensure robust statistical power and greater demographic representation.

In conclusion, our findings did not demonstrate a relationship between blood and hair mineral concentrations. However, the distinct patterns observed in hair mineral analysis reflect an extended temporal measurement interval of mineral status, in contrast to the point-in-time assessment provided by blood analysis. Blood tests for minerals represent the current (acute) levels of mineral status in the body, whereas hair mineral analysis captures chronic and long-term abnormalities in mineral levels. These two types of tests serve different clinical utilities: blood tests are useful for assessing the immediate status of mineral levels, while hair analysis offers insights into long-term trends and imbalances. Future studies with larger cohorts should further evaluate the role of hair mineral analysis in clinical settings. In addition to its potential utility in evaluating mineral status, hair analysis is already established as an efficient tool for detecting environmental exposures, including toxins such as pesticides, heavy metals, and polycyclic aromatic hydrocarbons [37–40]. Expanding this utility to the detection of long-term mineral imbalances or exposures will benefit patients and clinicians alike. Based on the results of this pilot study, we believe that a larger and more demographically diverse cohort of participants is needed to strengthen statistical reliability and enhance generalizability of the findings.

## Supplementary Information

Below is the link to the electronic supplementary material.

Supplementary file 1 (XLSX 17.3 KB)

## Data Availability

Data is provided within the manuscript or supplementary information files.
